# Editorial: Applied neuroimaging—Case report collection 2021

**DOI:** 10.3389/fneur.2022.1027252

**Published:** 2022-09-28

**Authors:** Jan Kassubek

**Affiliations:** Department of Neurology, University of Ulm, Ulm, Germany

**Keywords:** neuroimaging, multimodality, education, structure, function

The reports of single patients or very small patient cohorts as ‘case reports’ are *per se* not a part of systematic research and might thus be regarded as of limited clinico-scientific value. However, specific findings in rare conditions can be very educative to the readership in order to teach specific insights, on the one hand into clinical or technical aspects not explorable in larger groups due to their special features, or on the other hand into distinct pathophysiological relationships. In this *Frontiers in Neurology* Section for Applied Neuroimaging, i.e., a Section not defined by a disease entity but by a methodological application to various neurological diseases or conditions, such a collection of case reports has additional requirements: the cases should not show a mere imaging phenomenon, but should combine specific clinical and neuroimaging features that guide in the understanding of the given neurological condition or in the use of the imaging modality in the differential diagnostic or technical context, respectively. The ultimate aim, according to the Journal's mission statement, is to inform improvements in patient care.

The four case reports that were published fulfill these criteria very well and are excellent approaches to the aims summarized in the first paragraph. With respect to the understanding of brain changes associated with hypergravity, McGregor et al. investigated the intracranial fluid changes induced by spaceflight by a report of a crewmember who first experienced an aborted launch and then subsequently completed a 6-month International Space Station mission. As a participant of a prospective neuroimaging study, the subject received MRI scans before the aborted launch and once in addition afterwards, enabling a within-subject analysis to qualitatively compare the intracranial fluid changes following the aborted launch to those associated with the subsequent spaceflight, in comparison to a control group of 12 astronauts with completed ISS missions but without aborted launch. The investigation of this extremely specific condition demonstrated that exposure to hypergravity during launch and landing did not induce lasting intracranial fluid shifts given that after the launch abort, the subject's ventricular and free water changes were within the range of the control group, and that the fluid shifts after the launch abort were smaller and in the opposite direction compared to the same subject's brain changes after the subsequent spaceflight mission. The authors conclude that persisting spaceflight-induced intracranial fluid changes are therefore most likely due to prolonged microgravity exposure and/or other spaceflight factors.

In a case report which addresses a condition from clinical work of high current interest, Lin et al. report the case of a patient with coronavirus disease (COVID-19) vaccine-induced immune thrombotic thrombocytopenia (VITT) and the MRI-based diagnosis of sinus thrombosis in the left transverse sinus, sigmoid sinus, and left distal internal jugular vein who could be successfully treated by high-dose immunoglobulin and anticoagulation therapy, with no sequelae. The case highlights the role of neuroimaging in VITT management by the detection of brain pathology including sinus thrombosis. Two further reports demonstrated how other neuroimaging modalities than MRI contribute to the aims of the Research Topic. Dong et al. describe that C-arm cone-beam computed tomography (CBCT) was able to identify neovascularization, microcalcification, and plaque rupture in a patient's carotid artery. After carotid endarterectomy, histopathology confirmed that the high-density areas in CBCT represented neovascularization and macrocalcification with linear enhancement representing plaque rupture. The authors conclude that CBCT can be used as a promising supplement to current imaging modalities to evaluate plaque components more accurately. Finally, Ikeda et al. report a patient with Alzheimer's disease, carrying the homozygous APOE ε2 allele and presenting with recurrent lobar hemorrhages, cortical superficial siderosis, and immunohistochemically confirmed vascular amyloid β who was investigated by PET and ^18^F-THK5351 which is recognized as a dual-purpose compound that binds to both MAO-B and tau aggregates. By this approach, PET guided in the differentiation of the brain changes by identification of MAO-B concentrated regions, astroglial activation, secondary Wallerian tract degeneration, neuroinflammation due to cerebral amyloid angiopathy-related hemorrhages, and possible tau accumulation.

In summary, these case reports demonstrate how the neuroimaging-based analysis of single patients/subjects can provide an important contribution to clinicopathological and pathophysiological relationships in neurological diseases or other conditions (like hypergravity), further encouraging the—albeit strictly quality-controlled—publication of such casuistic findings.

## Author contributions

The author confirms being the sole contributor of this work and has approved it for publication.

## Conflict of interest

The author declares that the research was conducted in the absence of any commercial or financial relationships that could be construed as a potential conflict of interest.

## Publisher's note

All claims expressed in this article are solely those of the authors and do not necessarily represent those of their affiliated organizations, or those of the publisher, the editors and the reviewers. Any product that may be evaluated in this article, or claim that may be made by its manufacturer, is not guaranteed or endorsed by the publisher.

